# An adjuvanted inactivated murine cytomegalovirus (MCMV) vaccine induces potent and long-term protective immunity against a lethal challenge with virulent MCMV

**DOI:** 10.1186/1471-2334-14-195

**Published:** 2014-04-11

**Authors:** Huadong Wang, Yanfeng Yao, Chaoyang Huang, Xingxing Fu, Quanjiao Chen, Hongbo Zhang, Jianjun Chen, Fang Fang, Zhenyuan Xie, Ze Chen

**Affiliations:** 1 State Key Laboratory of Virology, Wuhan Institute of Virology, Chinese Academy of Sciences, Wuhan, 430071, Hubei, China; 2 College of Life Science, Hunan Normal University, Changsha 410081, Hunan, China; 3 Shanghai Institute of Biological Products, Shanghai 200052, China; 4 Graduate University of Chinese Academy of Sciences, Beijing 100049, China; 5 Xie Tu Community Medical Service Center, Xuhui, District of Shanghai, China

**Keywords:** Cytomegalovirus, Inactivated vaccine, Adjuvant, Long-term protective immunity

## Abstract

**Background:**

Human cytomegalovirus (HCMV) is a ubiquitous pathogen that causes serious problems in immunocompromised or immunologically immature hosts. Vaccination is the preferred approach for prevention of HCMV infection, but so far no approved HCMV vaccine is available. In this study, we assessed the immunogenicity and protective immunity of a formalin-inactivated murine cytomegalovirus vaccine (FI-MCMV) in a mouse model in combination with adjuvants MF59, alum, or chitosan.

**Methods:**

Specific-pathogen-free BALB/c mice aged 6–8 weeks were immunized twice, 3 weeks apart, with various doses of FI-MCMV (0.25 μg, 1 μg, 4 μg) with or without adjuvant. Mice were challenged with a lethal dose (5 × LD_50_) of a more virulent mouse salivary gland-passaged MCMV 3 weeks after the second immunization. The protective immunity of the vaccine was evaluated by determining the survival rates, residual spleen and salivary gland viral loads, body weight changes, and serum anti-MCMV IgG titers.

**Results:**

Immunization with FI-MCMV vaccine induced a high level of specific antibody response. Antigen sparing was achieved by the addition of an adjuvant, which significantly enhanced the humoral response to vaccine antigens with a wide range of doses. The level of live virus detected in the spleen on day 5 and in the salivary glands on day 21 after the lethal challenge was significantly lower in adjuvant-treated groups than in controls. Survival rates in adjuvant-treated groups also increased significantly. Furthermore, these protective immune responses were sustained for at least 6 months following immunization.

**Conclusions:**

These results show that inactivated MCMV vaccine is effective, and that the adjuvanted FI-MCMV vaccine provides more effective and longer-term protection than the adjuvant-free vaccine.

## Background

Human cytomegalovirus (HCMV), a member of the *Betaherpesvirinae* subfamily (type 5), is a ubiquitous pathogen that infects approximately 50–100% of the adult population worldwide, depending upon both socioeconomic factors and geographic location [1,2]. Cytomegalovirus is characterized by strict species specificity and a lifelong latency in the host [1]. Humans are the only reservoir of HCMV. In immunocompetent populations, the majority of HCMV infections present as asymptomatic or latent infections, but several high-risk groups, such as neonates, solid organ or allogeneic stem-cell transplant recipients, and HIV-infected patients, are susceptible to HCMV infection and may develop serious disease [3,4]. HCMV is the most common infectious cause of mental retardation and other birth defects in children, and causes extreme morbidity and mortality in congenitally infected newborns [5,6]. HCMV is also regarded as the most dangerous viral pathogen affecting transplantation. Therefore, CMV infection is indeed a serious public health problem [7,8].

Presently there is no suitable treatment for HCMV infection [9]. Therefore, effective prevention against this important pathogen is clearly desirable. The development of a vaccine to prevent HCMV infection has been assigned the highest priority by the US Institute of Medicine [7,10], but an approved HCMV vaccine is not yet available. Several candidate vaccines have been tested in clinical studies [11], including those based on live attenuated CMV, protein subunits, recombinant vectors, DNA, and peptides [4,10]. However, none of the experimental HCMV vaccines showed the desired protective efficacy in phase 3 clinical trials [2].

In the current study, we investigated the immunogenicity and protective efficacy of an inactivated CMV vaccine. Because of the strict species specificity of CMV infection, there is no animal model available for direct study of HCMV mechanisms employed during infection and immunity. Murine cytomegalovirus (MCMV) infection is the most widely used animal model simulating HCMV infection [12,13]. We therefore used a mouse model of lethal MCMV infection to study the immune response to an inactivated MCMV vaccine.

Adjuvants are non-specific immune-enhancing substances, of which there are multiple types. Currently, alum is the only adjuvant permitted for human use in the USA. Meanwhile many other adjuvants are being studied in animal experiments and human clinical trials [14-16], of which adjuvant MF59, an oil-in-water emulsion, has been shown to exhibit a safe and highly effective adjuvant activity. MF59 is a component of the flu vaccine FLUAD™, which has been used clinically in over 20 European countries [17]. Chitosan is a non-toxic, biologically tolerant and biodegradable natural polysaccharide isolated from exoskeletons of crustaceans or insects [18,19]. We designed our study to examine the protective effect of a vaccine with these three adjuvants.

## Methods

### Viruses and mice

We used the MCMV Smith strain and propagated the virus in NIH 3T3 cells. The MCMV derived from cell culture propagation is referred to as “TC-MCMV” (tissue culture-derived MCMV) [20]. The MCMV isolated from mouse salivary glands (SG) followed by *in vivo* passage for virulence enhancement is referred to as “SG-MCMV”, and was used in our lethal challenge experiments. High-virulence SG-MCMV was prepared from 10 *in vivo* passages. Passage-10 SG-MCMV stock had a titer on 3T3 cells of 10^7.1^ PFU/ml and a 50% lethal dose (LD_50_) of approximately 10^5^ PFU virus particles in BALB/c mice. Lethal challenge was performed with 5 × LD_50_ virus stock.

Specific-pathogen-free (SPF) female BALB/c mice, 6–8 weeks old, were purchased from the Center for Disease Control and Prevention in Hubei Province, China. They were bred in the Animal Resource Center at the Wuhan Institute of Virology, Chinese Academy of Sciences. All mice were maintained in SPF conditions. All procedures were reviewed and approved by the Animal Care Committee of Wuhan Institute of Virology.

### Preparation of inactivated vaccine and adjuvants

Inactivated vaccine was prepared with tissue culture-derived extracellular MCMV. Briefly, 3T3 cells were infected by MCMV and incubated at 37°C for 4–5 days. When the monolayer exhibited 100% cytopathic effect, the medium from the infected cells was harvested and the cell debris removed by centrifugation at 6,500 × *g* at 4°C for 20 min. The total volume of crude virus stock was quantified and inactivated by mixing with 37% formalin at a 1/4000 ratio, as described previously [21,22], except that inactivation was maintained at 4°C for 1 week with stirring. To confirm the absence of detectable infectivity, 100 μl of formalin-treated virus was used to infect the 3T3 cells. The 3T3 cells were incubated for 7 days, and no cytopathic effect was observed. Post-inactivation virus stock was pelleted and purified by ultracentrifugation, as described previously [12,23]. The resulting preparation was confirmed to be completely inactivated by plaque assay and intraperitoneal infection of BALB/c mice, in which we did not detect virus from the spleens, livers, and salivary glands of the mice. Mock formalin-inactivated (FI)-MCMV vaccine was prepared from the supernatants of uninfected NIH 3T3 cells and formalin treated as described above. The protein content of the inactivated whole-virion vaccine was determined with a Pierce BCA Protein Assay kit (Pierce, Rockford, IL), using BSA as a standard.

The adjuvants used were MF59, alum, and chitosan. MF59 is a submicron oil-in-water emulsion adjuvant, and its components include 5% squalene, 0.5% Tween 80, and 0.5% Span 85 (Sigma, St Louis, MO). Aluminum hydroxide (National Vaccine & Serum Institute, China) was added to the antigen solution to a final concentration of 0.5 μg/μl. Chitosan (Sigma) at a concentration of 0.2% (w/v) was diluted in a 25 mM sodium acetate solution (pH 5.0). The chitosan was prepared by: adding 0.04 g chitosan to 20 ml of a 0.1 M solution of glacial acetic acid [concentration 0.2% (w/v)], mixing it for about an hour to dissolve it, adjusting its pH with NaOH, and sterilizing it by filtration through a 0.45 μm filter. The final concentration of chitosan used for immunization was 50 μg/ml. The adjuvant and antigen were mixed and maintained at 4°C for 1 h and shaken to produce a homogenous mixture.

### Immunization of the mice

Each experimental group had 10 mice. The inactivated vaccines, at doses of 4 μg, 1 μg, and 0.25 μg with or without an adjuvant (MF59, alum, or chitosan), were prepared for immunization by intraperitoneal (i.p.) injection. Immunizations were performed twice, 3 weeks apart. The volume of each dose was 200 μl. The adjuvant-only group of mice was administered with MF59, alum, or chitosan only and the control group of mice received PBS. In addition, one group of mice was immunized with formalin-treated mock virus preparation (Mock). To test whether the FI-MCMV vaccine could provide long-term protection, immunizations were performed with 4 μg FI-MCMV and adjuvants, three times by i.p. injection.

To determine whether different immunization routes could provide similar protection, another experiment was performed as described above, except that the mice were immunized by intramuscular (i.m.) injection.

### Virus challenge

For lethal dose experiments, the more virulent passage-10 SG-MCMV was used. At 3 weeks after the second immunization, or 6 months after the third immunization, mice were challenged with a lethal dose (5 × LD_50_, 200 μl/mouse) of SG-MCMV by i.p. injection. This infection could cause systemic virus replication in mice and death of all unimmunized mice within 4–7 days after the infection. During the 3 weeks following virus challenge, body weight was recorded daily until death occurred.

### Detection of MCMV-specific antibody

Mouse serum samples were collected 3 weeks after the first and second immunizations. Serum samples were stored at -20°C. Anti-MCMV serum IgG titers were determined by enzyme-linked immunosorbent assay (ELISA). ELISA was performed in a 96-well polystyrene microtiter plate following standard protocols. Briefly, inactivated MCMV vaccine was added, followed by serial two-fold dilutions of sera from each group of mice and then biotin-conjugated goat anti-mouse IgG Ab (γ-chain specific) (Southern Biotechnology Associates, Birmingham, AL). Streptavidin conjugated with alkaline phosphatase (Southern Biotechnology Associates) was then added, followed by *p*-nitrophenyl-phosphate. Chromogen production was measured based on absorbance at 414 nm and 405 nm in an ELISA reader (Labsystems Multiskan Ascent Autoreader, Finland). Ab-positive cutoff values were set as mean + 2SD of unimmunized control sera. The ELISA Ab titer was expressed as the highest serum dilution to produce a positive reaction.

### Collection of samples

Five days after the virus challenge, four mice from each group were sacrificed and the spleens taken aseptically for detection of virus titer. The six remaining mice of each group were monitored for weight loss and survival. On day 21 post-challenge, when survival observations were complete, four mice were taken randomly from the remaining six mice for aseptic collection of salivary glands and the viral load was determined.

### Determination of the virus titer in infected organs

Spleens and salivary glands were homogenized in 1:10 (w/v) volume with minimal essential media containing 10% calf serum. The homogenized fluids were centrifuged and the supernatants stored in aliquots at -80°C.

Viral loads were determined using a plaque-forming cell assay. Briefly, organ homogenates were 10-fold serially diluted, and each dilution was used to infect the 3T3 cells cultured in 48-well plates. Infections were performed in triplicate and to each well was added viral dilution to 100 μl. After 1 h of absorption, supernatant was aspirated and 0.5 ml viscous medium was added to each well. After incubation for 4–6 days, viral plaques were counted and the viral PFU per milliliter were calculated. The virus titer for each experimental group was presented as the mean of mouse samples in that group ± SD.

### Statistical analysis

The experimental results were evaluated by One-Way ANOVA (SPSS 17.0 software for Windows). Test of homogeneity-of-variance followed by One-Way ANOVA with LSD post-hoc tests were used to test for differences among the same dose groups. If the *p*-value was less than 0.05, the difference was considered significant. The significance of the differences in survival rates between the experimental and control groups was determined using Fisher’s exact test.

## Results

### Antibody responses induced by FI-MCMV vaccine of various formulations

Preliminary experiments showed that immunizing mice intraperitoneally with 4 μg FI-MCMV vaccine three times protected mice against a lethal challenge of SG-MCMV, whereas two immunizations did not provide protection (data not shown). Based on these results, we sought to enhance vaccine effectiveness by using adjuvants. We used three doses (0.25 μg, 1 μg, and 4 μg) of FI-MCMV vaccine with or without an adjuvant (MF59, alum, or chitosan), with a booster shot 3 weeks after priming. Blood was taken 3 weeks after the first and second immunizations, and ELISA was used to determine serum anti-MCMV IgG titers (Table 1).

**Table 1:** **Antibody titers in sera of mice immunized with various doses of FI-MCMV with or without adjuvant by i.p. injection and viral loads in mouse organs after lethal viral challenge**^**a**^

**Immunogen**	**FI-MCMV**	**ELISA titer (2**^**n**^**) of IgG antibody**^**b**^		**Spleen virus titers (log**_**10 **_**PFU/ml)**	**Salivary gland virus titers (log**_**10 **_**PFU/ml)**
	**Dosage (μg)**	**Immunization once**	**Immunization twice**		
Adjuvant-free	0.25	3.33 ± 0.58	5.67 ± 0.52	5.16 ± 0.22	Not done^f^
+ Chitosan		5.83 ± 1.17^c^	9.83 ± 1.17^c^	5.12 ± 0.28^e^	Not done
+ Alum		6.67 ± 1.63^c^	11.17 ± 0.98^c^	5.06 ± 0.21^e^	Not done
+ MF59		7.50 ± 1.52^c^	12.83 ± 1.47^c,d^	4.06 ± 0.62^c,d,e^	4.76 ± 0.48
Adjuvant-free	1	5.33 ± 0.58	9.17 ± 0.98	5.03 ± 0.40^e^	Not done
+ Chitosan		6.67 ± 1.15	12.67 ± 1.03^c^	4.83 ± 0.12^e^	5.46 ± 0.57
+ Alum		7.83 ± 0.98^c^	14.00 ± 0.63^c^	4.31 ± 0.83^e^	5.12 ± 0.91
+ MF59		11.33 ± 0.82^c,d^	15.33 ± 1.03^c,d^	3.45 ± 0.56^c,e^	4.34 ± 0.42
Adjuvant-free	4	8.67 ± 0.82	11.67 ± 1.03	4.85 ± 0.21^e^	5.70 ± 0.38
+ Chitosan		10.50 ± 0.55^c^	14.83 ± 1.17^c^	3.95 ± 0.48^c,e^	4.57 ± 0.74^c^
+ Alum		11.00 ± 0.89^c^	15.17 ± 0.41^c^	3.61 ± 0.66^c,e^	4.10 ± 0.27^c^
+ MF59		12.17 ± 0.75^c^	17.00 ± 1.10^c,d^	2.08 ± 0.81^c,d,e^	2.31 ± 0.43^c,d^
Chitosan only	-	Undetected	Undetected	5.76 ± 0.29	Not done
Alum only		Undetected	Undetected	5.83 ± 0.38	Not done
MF59 only		Undetected	Undetected	5.74 ± 0.18	Not done
Mock		Undetected	Undetected	5.61 ± 0.36	Not done
PBS		-	-	5.94 ± 0.25	Not done

^a^The mice were immunized by i.p. injection twice (3 weeks apart) with various doses of vaccine (0.25, 1, or 4 μg) with or without adjuvant (MF59, alum, or chitosan). Serum samples from immunized mice were obtained 3 weeks after the first and second immunization, respectively. Mice were challenged with a lethal dose of SG-MCMV 3 weeks after the boost. Spleen virus titers 5 days later and salivary gland virus titers 3 weeks after the challenge were measured. Results are expressed as means ± standard deviation of tested mice in each group.

^b^Serum samples were diluted by two-fold serial dilutions and “n” represents the dilution factor.

^c^Significant difference (*p* < 0.05), compared with the corresponding adjuvant-free subjects.

^d^Significant difference (*p* < 0.05), compared with the corresponding alum adjuvant subjects.

^e^Significant difference (*p* < 0.05), compared with control subjects.

^f^Viral titer measurement was not performed because of the mouse death before the day 21 post-infection.

The FI-MCMV vaccine had good immunogenicity and was able to induce a humoral immune response. Antibody titers after the immunization boost were higher than those after priming. IgG antibody levels correlated positively with the vaccine dose. The antibody titer was greater and the elicited immune response stronger with a higher vaccine dose. When the vaccines were formulated with an adjuvant, the antibody levels increased significantly compared with adjuvant-free vaccines. No anti-MCMV IgG was detected in MF59, alum, and chitosan alone or in mock vaccine-treated mice, which suggested that neither the adjuvant-only treatment nor the Mock vaccine could induce specific anti-MCMV immune responses.

Antibody titers in the alum-adjuvanted group were about 10 times higher than those in the corresponding vaccine-only groups. Antibody titers in the chitosan group were close to those in the alum group. Interestingly, MF59-adjuvanted groups had the best antibody response. These results suggest that MF59 is a more effective adjuvant than alum and is capable of significantly enhancing the humoral immune response.

### Protection provided by immunization with FI-MCMV vaccine alone or with adjuvants against lethal virus challenge

Mice were immunized twice, with a 3-week interval, with FI-MCMV alone or with adjuvants. Three weeks after the boost, mice were challenged with a lethal dose of SG-MCMV (5 × LD_50_). Table 2 shows the survival rates for all the experimental groups.

**Table 2:** **Survival after lethal SG-MCMV challenge in mice immunized intraperitoneally with various doses of inactivated vaccine with or without adjuvant**^**a**^

**Immunogen**	**Protection against SGV challenge (Survival mice/total mice) by various doses of FI-MCMV immunization**			
	**-**	**0.25 μg**	**1 μg**	**4 μg**
Adjuvant-free	0/6	0/6	0/6	2/6
+ Chitosan	0/6	0/6	2/6	6/6^*^
+ Alum	0/6	0/6	4/6	6/6^*^
+ MF59	0/6	5/6^*^	6/6^*^	6/6^*^
Mock	0/6	-	-	-
PBS	0/6	-	-	-

^a^The mice were immunized twice, 3 weeks apart, with various doses (0.25, 1, or 4 μg) of the vaccine with or without adjuvant (MF59, alum, or chitosan) by i.p. injection. The mice were challenged with a lethal dose of SG-MCMV 3 weeks after the second immunization. Mouse survival 3 weeks after challenge was measured.

*Significant difference (*p* < 0.05), compared with control subjects.

All mice in the control group (including mock vaccine) and adjuvant-alone treatment groups died within 8 days after a lethal challenge with SG-MCMV. Immunization with FI-MCMV vaccine (no adjuvant) at all three doses did not provide sufficient protection, but immunization with adjuvanted FI-MCMV significantly improved protection. At a low dose (0.25 μg), the survival rate in the FI-MCMV plus MF59 group reached 83.3%, while mice immunized with FI-MCMV plus alum or chitosan were not protected. At the 1 μg dose, all mice in the FI-MCMV plus MF59 group survived, while mice in the alum- or chitosan-adjuvanted groups were not completely protected. At the 4 μg dose, all three adjuvanted groups had full protection. These results indicated that immunizing twice with adjuvanted FI-MCMV vaccine could achieve excellent protection.

### Infectious viral loads in mouse spleen and salivary glands after lethal challenge

The spleen is a key organ of virus invasion and replication at the acute phase of SG-MCMV infection, and viral load in the spleen decreases rapidly after the acute phase. The salivary gland is another important organ for CMV infection, latency, and dissemination. The capacity of vaccine-induced protective immunity to eradicate infected viruses *in vivo* may be directly reflected by the titers of infectious viruses in the spleen and salivary glands. Spleen and salivary gland samples were collected on days 5 and 21 after the lethal challenge, respectively.

As shown in Table 1, the titers of virus in the spleen for the control mice after challenge reached 10^5.94^ PFU/ml, whereas the titers in the spleen following vaccination decreased greatly. Addition of an adjuvant (MF59, alum, or chitosan) to vaccines reduced the virus titers in the spleen. The MF59-adjuvanted groups had significantly lower titers than the vaccine-only group for all three doses, while the alum- and chitosan-adjuvanted groups had significantly lower titers, though only for the 4 μg dose, than the vaccine-only group. These results clearly show that immune responses induced by adjuvanted-inactivated vaccine can effectively eradicate infectious virus from the spleen. The most dramatic difference was observed in the MF59-adjuvanted 4 μg vaccine group, where the virus titer decreased more than 7000-fold relative to controls.

Salivary gland viral loads were determined only on surviving mice since mice with no or little protection died. Mice immunized with adjuvanted vaccine had lower salivary gland titers than mice in the vaccine-only group (Table 1). Moreover, the MF59-adjuvanted 4 μg vaccine group had significantly lower titers than the corresponding alum- or chitosan-adjuvanted groups. The salivary gland is widely believed to be the most important organ for CMV latency and dissemination. Therefore, a significantly lower salivary gland viral load is an important indicator of the protective effect of a vaccine.

### Signs of infection and body weight change in mice after lethal challenge

The challenge experiment found that morbidity was observed at day 2 after the challenge, and within a week, obvious weight losses were observed in unprotected mice. The main signs of infection included lethargy, piloerection, hunched posture, and emaciation. Unprotected mice typically died within 4–8 days of infection. In contrast, mice with protective immunity usually started to recover gradually after 1 week and did not present with visible signs of infection for up to 2 weeks after infection.

**Figure 1:**
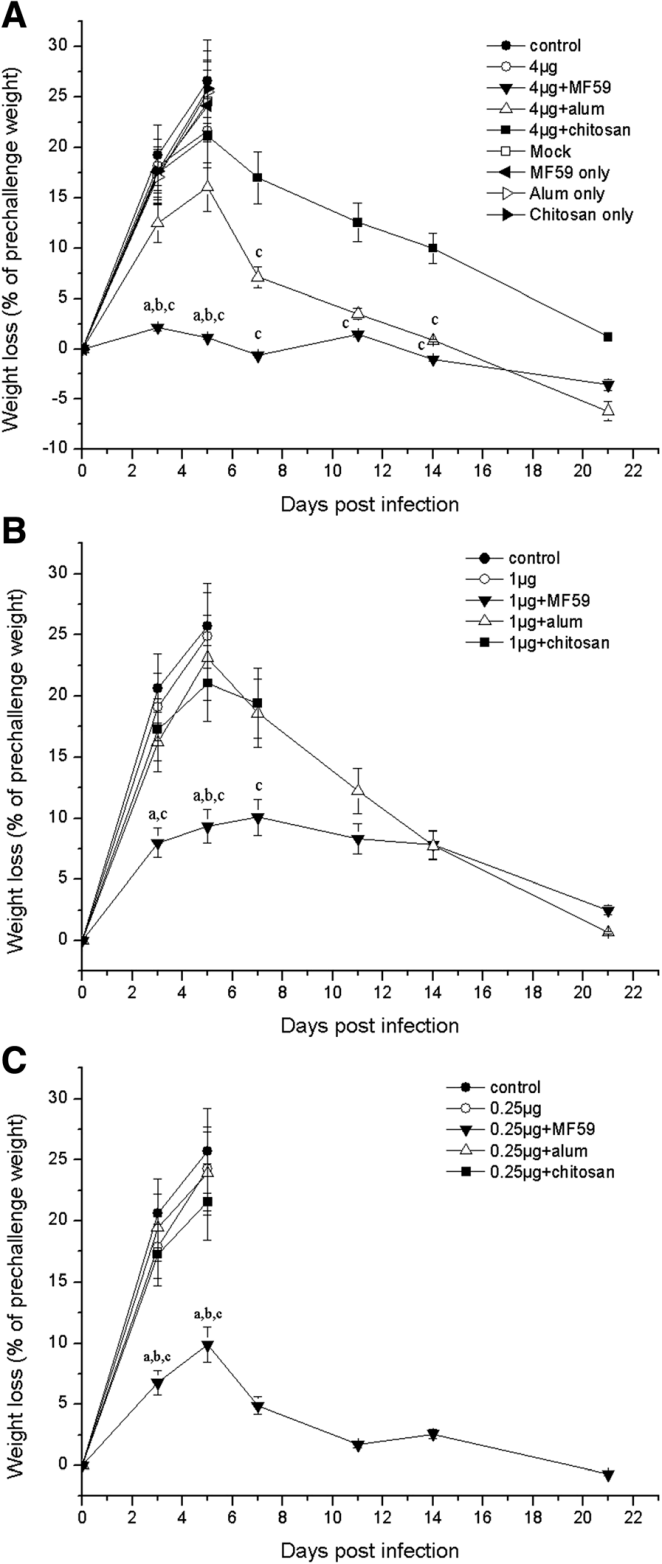
**Bodyweight changes after virus challenge.** Mice were intraperitoneally immunized twice, at a 3-week interval, with 4 μg **(A)**, 1 μg **(B)**, and 0.25 μg **(C)** of the vaccine with or without an adjuvant (MF59, alum, or chitosan). Mice were challenged with a lethal dose of SG-MCMV 3 weeks after the second immunization and the body weights were measured from the time of the challenge to 3 weeks after the challenge. Data points represent mean ± SD. ^a^Significant difference (*p* <0.05) vs the corresponding adjuvant-free subjects. ^b^*p* <0.05 vs the corresponding alum-adjuvant subjects. ^c^*p* <0.05 vs the corresponding chitosan-adjuvant subjects.

The body weight changes were observed for 21 days after the challenge (Figure 1). Weight loss was most marked by day 5, and the largest weight loss in the control group was close to 30%. In contrast, body weight loss in all immunized groups was relatively reduced, indicating protection provided by vaccination. The weight loss in the 4 μg dose groups was lower than that in the mice given 1 μg and 0.25 μg doses. Mice immunized with adjuvanted vaccines showed apparently more subtle signs of infection and smaller weight loss ratios, and their body weight recovery was faster than the vaccine-only groups.

When the three adjuvants were compared for protection against the challenge, MF59 showed superior results relative to alum and chitosan. However, 4 μg FI-MCMV produced full protection in all three adjuvanted groups, although morbidity differed among mice in these groups. Mice immunized with 4 μg and 1 μg doses of MF59-adjuvanted vaccine showed little signs of infection, and the 4 μg FI-MCMV/MF59 showed almost no weight loss. In contrast, mice immunized with alum- or chitosan-adjuvanted vaccine, with even the highest dose, had apparent signs of infection within 1 week of challenge combined with evident weight loss.

### Immunization of mice with adjuvanted FI-MCMV could provide long-term protection against lethal challenge

To determine whether immunization with FI-MCMV could provide long-term protection for mice, we immunized mice by i.p. injection with 4 μg FI-MCMV vaccine with or without an adjuvant (MF59, alum, or chitosan) three times at 3-week intervals. Six months after the third immunization, the mice were challenged with a lethal dose of SG-MCMV, and the survival rates observed for 3 weeks (Table 3).

**Table 3:** **Long-term protection in mice immunized with inactivated vaccine with or without adjuvant**^**a**^

**Immunogen**	**Dosage (μg)**	**ELISA titer (2**^**n**^**) of IgG antibody**^**b **^**(6 months after the last immunization)**	**Protection against SGV challenge**
			**Survival mice/total mice**
Adjuvant-free	4	8.0 ± 0.8	3/14
+Chitosan		9.5 ± 1.0^d^	6/14^c^
+Alum		11.7 ± 0.5^d^	12/14^c,d^
+MF59		12.3 ± 0.6^d^	13/14^c,d^
PBS	-	-	1/15

^a^Mice were immunized by i.p. injection twice, 3 weeks apart, with 4 μg of the inactivated vaccine with or without an adjuvant (MF59, alum, or chitosan). Mice were challenged with a lethal dose of SG-MCMV 6 months after the last immunization. Serum samples from the immunized mice were collected 2 days prior to challenge. The survival of mice 3 weeks after the challenge was measured.

^b^Values represent means ± SD of each group.

^c^Significant difference (*p* < 0.05), compared with control.

^d^Significant difference (*p* < 0.05), compared with the corresponding adjuvant-free subjects.

Control mice started to die at day 3 post-challenge, and by day 6 only 1/15 mice was still alive. The survival rate of mice immunized with 4 μg FI-MCMV alone was 21.4%—a sharp contrast to the full protection provided by three immunizations observed in preliminary experiments. This suggested that protective immunity gradually declined with time, which was consistent with pre-challenge serum anti-MCMV IgG titers. Antibody levels at 6 months declined for immunized groups, to various degrees, although antibody levels in the three adjuvanted groups remained at levels significantly higher than those in the vaccine-only groups (Table 3).

The long-term protection provided by the three adjuvanted vaccines was compared. When challenged with a lethal dose of SG-MCMV at 6 months post-immunization, MF59-treated mice were protected, with a survival rate of 93% (13/14). The alum-adjuvanted group had a survival rate of 86% (12/14). In comparison, chitosan had a less satisfactory adjuvant effect, with a survival rate of 43% (6/14). These results strongly suggest that immunization with adjuvanted FI-MCMV vaccine elicits a stronger protective immune response and can provide longer protection than immunization with adjuvant-free vaccine. From the perspective of long-term protection, adjuvant MF59 had similar efficacy as Alum, both of which were slightly superior to chitosan. However, all conferred significantly better protection than the adjuvant-free vaccine.

### Protection against lethal SG-MCMV challenge in mice immunized with FI-MCMV vaccine alone or adjuvanted FI-MCMV by intramuscular injection

In the above-described experiments, the vaccine formulation was applied intraperitoneally. To mimic real-life settings, we also performed vaccinations by i.m. injection, which is a common route in vaccine administration. Mice were immunized with various doses of FI-MCMV intramuscularly with or without MF59, alum, or chitosan as adjuvant. Meanwhile some other mice were treated with PBS or the three adjuvants only (Table 4). Serum samples from the immunized mice were obtained 3 weeks after the first and second immunization and used for IgG Ab assays. Three weeks after immunization, all the mice were challenged with a lethal dose of SG-MCMV.

**Table 4:** **Antibody titers and protection against SG-MCMV challenge in mice immunized with various doses of FI-MCMV with or without adjuvant by i.m. injection**^a^

**Immunogen**	**FI-MCMV**	**ELISA titer (2**^**n**^**) of IgG antibody**^**b**^		**Protection against SG-MCMV challenge**	
	**Dosage (μg)**	**Immunization once**	**Immunization twice**	**Spleen virus titers (log**_**10 **_**PFU/ml)**	**Survival mice/tested mice**
Adjuvant-free	0.25	3.67 ± 1.15	5.33 ± 0.58	5.27 ± 0.19	0/6
+ Chitosan		5.00 ± 1.00	9.67 ± 1.15^c^	5.08 ± 0.13	0/6
+ Alum		6.33 ± 1.53^c^	11.33 ± 1.15^c^	5.14 ± 0.11	0/6
+ MF59		7.33 ± 0.58^c^	12.33 ± 1.53^c^	4.58 ± 0.36^c,e^	2/6
Adjuvant-free	1	4.67 ± 1.15	10.00 ± 1.73	5.13 ± 0.29	0/6
+ Chitosan		6.33 ± 0.58	12.00 ± 1.00^c^	4.64 ± 0.50^e^	2/6
+ Alum		7.33 ± 0.58^c^	12.33 ± 1.15^c^	4.78 ± 0.15^e^	1/6
+ MF59		10.67 ± 1.15^c,d^	15.00 ± 1.00^c,d^	3.51 ± 0.49^c,d,e^	5/6^e^
Adjuvant-free	4	7.67 ± 0.58	11.67 ± 1.52	4.41 ± 0.48^e^	3/6
+ Chitosan		10.00 ± 1.73^c^	13.67 ± 0.58^c^	4.02 ± 0.51^e^	5/6^e^
+ Alum		10.33 ± 0.58^c^	14.00 ± 1.00^c^	3.80 ± 0.11^c,e^	6/6^e^
+ MF59		11.67 ± 0.58^c^	16.33 ± 0.58^c,d^	2.56 ± 0.77^c,d,e^	6/6^e^
Chitosan only	-	Undetected	Undetected	5.87 ± 0.17	0/6
Alum only		Undetected	Undetected	5.68 ± 0.27	0/6
MF59 only		Undetected	Undetected	5.62 ± 0.38	0/6
Mock		Undetected	Undetected	5.73 ± 0.34	0/6
PBS		-	-	5.91 ± 0.39	0/6

^a^The mice were immunized twice (3 weeks apart) with various doses of vaccine (0.25, 1, or 4 μg) with or without adjuvant (MF59, alum, or chitosan) by i.m. injection. Serum samples from immunized mice were obtained 3 weeks after the first and second immunization, respectively. Three weeks after immunization, all the mice were challenged with a lethal dose of SG-MCMV. Spleen virus titers 5 days after challenge and survival rates of mice 21 days post-infection were determined. Results are expressed as means ± SD of tested mice in each group.

^b^Serum samples were diluted by two-fold serial dilutions and “n” represents the dilution factor.

^c^Significant difference (*p* < 0.05), compared with the corresponding adjuvant-free subjects.

^d^Significant difference (*p* < 0.05), compared with the corresponding alum adjuvant subjects.

^e^Significant difference (*p* < 0.05), compared with corresponding control subjects.

As shown in Table 4, the FI-MCMV vaccine also effectively induced a humoral immune response when given intramuscularly. Antibody titers after the booster injection were higher than those after priming. After immunization with adjuvanted vaccines, antibody levels increased obviously compared with immunization with adjuvant-free vaccines. MF59-adjuvanted groups showed the best antibody response. Antibody titers in the alum groups were close to those in the chitosan groups. No anti-MCMV IgG was detected in MF59-, alum-, or chitosan-only treated mice, and these mice died within 1 week after lethal challenge. Vaccination with FI-MCMV alone at all three doses did not provide effective protection, but immunization with adjuvanted FI-MCMV obviously improved protection. At the 4 μg dose only, full protection was observed in all three adjuvanted groups by i.m. route. At the 1 μg dose, the survival rates in the adjuvanted FI-MCMV groups decreased correspondingly. No effective protection was observed in the mice immunized with adjuvanted vaccine at the low 0.25 μg dose. Vaccinated groups had lower virus titers in the spleen than the control and adjuvant-only groups. Addition of an adjuvant (MF59, alum, or chitosan) to vaccines further reduced virus titers in the spleen. The most remarkable difference was found in the MF59-adjuvanted 4 μg vaccine group, as its spleen virus titer was almost 10^3^-fold lower than that of the control group (Table 4). This i.m. immunization experiment also illustrated that adjuvanted FI-MCMV vaccine induced better immune protection and MF59 showed a superior immune enhancement effect relative to alum and chitosan.

## Discussion

Several features of HCMV infection render the research and development of a safe and effective CMV vaccine quite difficult. CMV is able to evade the immune response, and some HCMV genes modulate host innate or adaptive immune responses to benefit survival of the virus itself [24]. Studying the immune-evading mechanisms of CMV is critical for the development of an effective CMV vaccine [1]. In addition, the lifelong latency and ability to re-infect, despite pre-existing natural immunity, represent a significant safety concern for the application of attenuated live vaccines [4,16]. CMV has strict species specificity, and therefore no animal model exists that would allow the direct study of the mechanisms of HCMV infection and the effects of vaccine immunization [25]. All of the above factors have restricted the progress of CMV vaccine development. Despite many attempts, no CMV vaccine has been approved for human use [4,10].

The essential components that constitute an effective CMV vaccine are not yet clear [26,27]. There is increasing awareness that the actual host immune responses for HCMV infection are far more complex than was previously imagined. Considering that the protective effects for most CMV vaccine candidates in clinical studies are unsatisfactory, the singular vaccine antigen composition might be a key reason. Therefore, some experts have pointed out that the immune responses induced by a safe and effective CMV vaccine should target multiple antigens expressed at different stages of virus replication, and more importantly, vaccine effectiveness should not be impaired by the virus evading the immune response [4]. Another important issue to consider is the clinical settings of reinfection by different CMV strains, a reminder that vaccine design should aim for cross-protection against infection by multiple epidemic CMV strains. Inactivated CMV vaccines with whole virus particle proteins, especially those envelope glycoproteins critical for immunity, are capable of inducing more diverse immune responses, which might be more similar to the host immune responses induced by natural infections. In contrast, a subunit vaccine has a singular antigenic component, and its limited antigen epitopes could not exert the same role in inducing host immune responses as multiple antigen epitopes of an inactivated vaccine under near natural conditions. Therefore, it is necessary to study the immunization effects and strategies of inactivated CMV vaccines. For the past 30 years researchers have studied the inactivated MCMV vaccine in animal experiments. In 1980, Tolpin et al. reported that two immunizations of mice with inactivated MCMV vaccine could provide a protection rate of 89% against a low-level virus challenge. However, most mice still developed mild or subclinical infections after challenge [21]. In 1996, Geoffroy et al. demonstrated that the protective rate of an MCMV vaccine inactivated by sodium periodate or β-propiolactone reached 100%, but the long-lasting protection offered was incomplete [22]. In 2002, Morello et al. proved that FI-MCMV adjuvanted with alum induced effective protection; however, they only performed a sub-lethal challenge (<1 × LD_50_) [3]. Therefore, development of an inactivated vaccine that could provide protection against lethal challenge and long-term immunity remains to be a difficult task.

In addition to vaccines, adjuvant and delivery protocols must be optimized. Co-administering vaccine with a suitable adjuvant could significantly enhance the immune response. These observations have prompted searches for novel, safe, and effective adjuvants [28]. Many studies have reported that adjuvants may enhance the immunogenicity of inactivated or subunit vaccines, such as those for influenza or HIV [29-31]. Here, we sought to immunize mice with an inactivated CMV vaccine and several different adjuvants to test the effects of adjuvant enhancement.

Our results illustrated that inactivated CMV vaccine demonstrated good immunogenicity. Primary and booster immunizations of 4 μg vaccine plus any of the three adjuvants provided full protection. In particular, the MF59 adjuvant, even at a low dose of 0.25 μg by i.p. injection, conferred excellent protection against lethal challenge for the mice. These results illustrated that vaccine plus adjuvant could provide better protection, allowing for a reduction in the number of immunizations and sparing the antigen dose. When the vaccine was given intramuscularly, which is the most common route used to administer adjuvanted vaccines in real-life settings, the results were similar to those obtained by i.p. injection, whereas the overall protective efficacy of vaccine administered intramuscularly was slightly inferior to that administered intraperitoneally. The minimum vaccine dose of FI-MCMV for conferring complete protection for the mice was higher for an i.m. immunization than that for an i.p. immunization.

When the three types of adjuvants are compared for immune enhancement, MF59 is the most effective, and chitosan has equivalent activities as alum. The mechanisms by which these three adjuvants work are not clear [32]. Alum adjuvant may granulate antigens and activate the innate immune pathway, creating an active immune environment at the injection site, and thus promoting the co-immunized antigen to induce an antibody response [17,33,34]. Adjuvant MF59 is a type of oil-in-water emulsion, in which the oil drops consist of metabolizable squalene and the emulsifying agent is a mixture of Tween 80 and Span 85. Animal experiments have demonstrated that MF59 differs from alum in that it does not prolong antigen presence at the injection site, indicating that MF59 does not exert its adjuvant effect through the “depot-effect” [31]. Studies have shown that at least three human cells are targets of MF59, including monocytes, macrophages, and granulocytes. MF59 induces monocytes to differentiate towards dendritic cells by up-regulating the co-stimulatory molecule CD86 and down-regulating the monocyte marker CD14. MF59 induces a series of *in vivo* effects, such as promoting antigen uptake, chemokine release, and cell differentiation, and significantly promotes a strong immune response to co-administered antigens by inducing a local inflammatory environment at the injection site [17,35,36]. Chitosan is a safe, non-toxic, natural cationic polymer. Its advantages include a relatively low price, good tolerability, auto-degradability, and high viscosity, which have made it an ideal adjuvant candidate. Chitosan can adsorb to negatively charged substances, such as cell membranes and mucosa surfaces, and can prolong the half-life of antigens on the mucosal surface. In addition, chitosan can stimulate macrophages and natural killer cells and therefore promote immune responses [37]. Both MF59 and chitosan are considered to have excellent prospects as adjuvants.

There is increasing evidence that a reduction in viral load provides significant therapeutic benefits to patients suffering from HCMV disease, and that CMV vaccination is the most practical approach for realizing this goal [38]. In our experiments, the viral loads in both spleen and salivary glands for immunized mice were significantly lower than those for the control mice. For example, the virus titer in the spleen of the MF59-adjuvanted group (4 μg) was more than 10^3^-fold lower than that of the control group, indicating that immunity induced by inactivated vaccine could effectively eradicate infected viruses *in vivo*. The reduction of viral load in target organs such as the salivary glands and spleen is an important indicator for demonstrating the protective effects of a CMV vaccine.

As CMV vaccines currently in development do not prevent viral infection, several scholars have suggested that a more realistic goal for CMV vaccines should be to limit or prevent HCMV disease rather than to entirely prevent infection [4,38]. Our study supports this perspective. Although immunization with an inactivated vaccine, in particular with adjuvants, elicited a robust immune response and results in significantly lower virus titers in infected organs compared with controls, it could not achieve complete viral clearance *in vivo*. We found only one instance of virus being undetectable (in a mouse salivary gland in the MF59-adjuvanted group). Certainly, we need to consider that the virus used for challenge had enhanced virulence via serial passage of SG homogenates and was much more virulent than wild-type virus. The challenge method used was a lethal high-dose infection, and thus the experimental challenge was far more hazardous than would occur naturally. Therefore, it is entirely possible that an inactivated CMV vaccine could elicit better immune protection against naturally occurring common CMV infections.

Some studies have highlighted that the duration of protective immunity induced by CMV vaccines is also an important factor in vaccine development [4]. People are mostly interested in a vaccine that can prevent congenital infection or disease, and has the capacity to maintain long-term immunity, lasting for several years. One method of inducing long-term immunity is to adopt multiple regular boosts, but the shortcoming of such a method is increased cost. Alternatively, adjuvant can be used to enhance immune responses to achieve satisfactory immune protection.

## Conclusions

Our study demonstrates that inactivated CMV vaccine has good immunogenicity and is a feasible candidate for preventing CMV infection. Co-administration of the vaccine with adjuvant significantly improves the efficacy of the inactivated vaccine and enhances the immune response in mice. More importantly, immunization with adjuvanted vaccine may provide more effective and longer-term protection than adjuvant-free vaccines.

## Abbreviations

MCMV: Murine cytomegalovirus; HCMV: Human cytomegalovirus; FI-MCMV: Formalin inactivated murine cytomegalovirus vaccine; SG-MCMV: Mouse salivary gland passaged MCMV; Alum: Aluminum hydroxide; TC-MCMV: Tissue culture-derived MCMV; SPF: Specific pathogen free; i.p.: Intraperitoneal injection; i.m.: Intramuscular injection; ELISA: Enzyme-linked immunosorbent assay.

## Competing interests

The authors declare that they have no competing interests.

## Authors’ contributions

HDW did most of the experimental work and drafted the manuscript. YFY, CYH, XXF, and ZYX participated in the analysis of humoral responses and plaque assays. QJC, HBZ, FF, and JJC participated in the immunization of mice. ZC revised the manuscript for important intellectual content and gave final approval of the version to be published. All authors read and approved the final manuscript.

## Pre-publication history

The pre-publication history for this paper can be accessed here:

http://www.biomedcentral.com/1471-2334/14/195/prepub
